# Efficacy and safety of tacrolimus monotherapy versus cyclophosphamide–corticosteroid combination therapy for idiopathic membranous nephropathy: A meta-analysis

**DOI:** 10.1097/MD.0000000000026628

**Published:** 2021-07-16

**Authors:** Lifeng Gong, Min Xu, Wei Xu, Weigang Tang, Jingkui Lu, Wei Jiang, Fengyan Xie, Liping Ding, Xiaoli Qian

**Affiliations:** a Department of Nephrology, Wujin Hospital Affiliated with Jiangsu University, Changzhou, Jiangsu, China; b Department of Nephrology, The Wujin Clinical College of Xuzhou Medical University Changzhou, Jiangsu, China.

**Keywords:** cyclophosphamide, idiopathic membranous nephropathy, meta-analysis, tacrolimus

## Abstract

**Objective:**

The objective of this meta-analysis was to compare the efficacy and safety of tacrolimus (TAC) monotherapy versus cyclophosphamide (CTX)-corticosteroid combination therapy in idiopathic membranous nephropathy (IMN) patients.

**Methods:**

Databases including the PubMed, Embase, the Cochrane Library, China National Knowledge Infrastructure, and Wanfang databases were searched from inception to October 20, 2020. Eligible studies comparing TAC monotherapy and CTX-corticosteroid combination therapy in IMN patients were included. Data were analyzed using Review Manager Version 5.3.

**Results:**

Nine studies were included in the meta-analysis. One randomized controlled trial and eight cohort studies involving 442 patients were identified. Compared with CTX-corticosteroid combination therapy for IMN, TAC monotherapy had higher complete remission (CR) at month 6 (odds ratio [OR] 2.18, 95% confidence interval [CI] 1.35–3.50, *P* < .01). The 2 therapeutic regimens had similar partial remission (OR 0.69, 95% CI 0.45–1.04, *P* = .08), total remission (OR 1.38, 95% CI 0.85–2.23, *P* = 0.19) at month 6, and similar CR (OR 1.64, 95% CI 0.84–3.19, *P* = .15), partial remission (OR 0.71, 95% CI 0.37–1.38, *P* = 0.31), and total remission (OR 1.29, 95% CI 0.55–3.01, *P* = .56) after 1 year. The relapse rate of the TAC group was higher than that of the CTX group, but the difference was not statistically significant (OR 1.85, 95% CI 0.75–4.53, *P* = .18). There was no difference between the 2 therapeutic regimens concerning glucose intolerance (OR 1.15, 95% CI 0.61–2.14, *P* = .67), acute renal failure (OR 1.14, 95% CI 0.39–3.33, *P* = .81), or tremors (OR 4.39, 95% CI 0.75–25.67, *P* = .10). Incidences of gastrointestinal symptoms (OR 0.29, 95% CI 0.10–0.79, *P* = .02), infection (OR 0.18, 95% CI 0.08–0.39, *P* < 0.01), leukopenia (OR 0.14, 95% CI 0.04–0.51, *P* < .01), and abnormal aminotransferase (OR 0.31, 95% CI 0.13–0.77, *P* = .01) in the TAC group were all lower than those in the CTX group. Subgroup analysis showed that there was no significant difference between the TAC group and the CTX combined with corticosteroid 0.8 to 1 mg/kg/day group concerning CR at month 6 (*P* > .05). There was no significant difference between the TAC group and the CTX combined with corticosteroid 0.5 mg/kg/day group concerning abnormal aminotransferase (*P* > .05).

**Conclusion:**

TAC monotherapy is comparable to CTX-corticosteroid combination therapy for renal remission in IMN patients. TAC monotherapy had a higher CR in the early stage and had fewer drug-related adverse effects. The relapse rate of TAC monotherapy was higher than that of CTX-corticosteroid combination therapy, but the difference was not significant.

## 1 Introduction

Idiopathic membranous nephropathy (IMN) is regarded as one of the most common causes of nephrotic syndrome in adults.^[1–3]^ Among IMN patients with persistent nephrotic syndrome, approximately 30% to 40% will progress to end-stage renal disease within 10 years.^[4–6]^ Cyclophosphamide (CTX) combined with corticosteroids has been recommended as an initial therapy for IMN according to Kidney Disease Improving Global Outcomes.^[7]^ However, the significant drug-related adverse effects of this standard therapy limit its administration in some patients.^[8–10]^

Cyclosporine (CsA) and tacrolimus (TAC) are recommended as alternative therapy regimens for IMN.^[11]^ Compared to cyclosporine, TAC showed a stronger immunosuppressive effect and fewer side effects.^[12–15]^ Some meta-analyses showed that TAC combined with corticosteroids also had a satisfactory effect for IMN compared with CTX combined with corticosteroids. However, corticosteroids still exhibit adverse effects.^[16,17]^ In recent years, some studies have compared TAC monotherapy with CTX combined with corticosteroids for IMN concerning efficacy and safety, and the results are controversial. Our meta-analysis was conducted to compare the efficacy and drug safety between TAC monotherapy with CTX combined with corticosteroids for IMN.

## 2 Materials and methods

### 2.1 Search strategy

Our meta-analysis has been reported in line with the Preferred Reporting Items for Systematic Reviews and Meta-Analyses and Assessing the Methodological Quality of Systematic Review Guidelines. Our meta-analysis was registered at the International Prospective Register of Systematic Reviews (Registration number: CRD42020211061). Ethical approval was not necessary because our study was a statistical analysis.

We searched the PubMed, Embase, the Cochrane Library, China National Knowledge Infrastructure, and Wanfang databases from inception to October 20, 2020. The combined text and MeSH terms included idiopathic membranous nephropathy, cyclophosphamide, and tacrolimus. In addition, the cited papers and relevant references were searched manually to identify eligible studies. There were no language restrictions.

### 2.2 Inclusion criteria

The inclusion criteria were defined as follows:

1. Randomized controlled trials (RCTs), cohort, or case–control studies;
2. IMN patients with nephrotic syndrome and serum creatinine level of <133 μmol/L;
3. Studies designed to compare TAC monotherapy with a CTX-corticosteroid combination therapy;
4. The main endpoints of the review were partial remission (PR), complete remission (CR), and total remission (TR). The secondary endpoints were relapse and drug-related adverse effects.

### 2.3 Exclusion criteria

The exclusion criteria were defined as follows:

1. Case series, commentaries, and reviews;
2. Lack of relevant outcomes data;
3. Patients with secondary membranous nephropathy, malignant tumor, infection (hepatitis B or C virus infection, tuberculosis, and syphilis), diabetes mellitus, pregnancy or lactation, and active gastrointestinal bleeding.

### 2.4 Data extraction and quality assessment

Data were extracted independently by 2 investigators using standard data extraction forms. In the case of disagreement, a third investigator was consulted. We extracted characteristics including first author, the year of publication, location, study design, follow-up period, age, sex, sample size, specific drug treatment program, and all the outcomes (definitions of CR, PR, and relapse are shown in Table 1**)**. TR was defined as either CR or PR. Relapse was defined as proteinuria >3.5 g/day in patients who had achieved CR or PR. The Cochrane assessment tool was used to evaluate the quality of RCTs.^[18]^ The Newcastle–Ottawa scale (NOS) was used to evaluate the quality of nonrandomized studies.^[19]^

**Table 1:** Definition of complete remission,partial remission and relapse.

Study	Complete remission	Partial remission	Relapse
Wang^[20]^	Proteinuria <0.3 g/day with normal serum ALB (>35 g/L) and renal function	Proteinuria 0.3–3.5 g/day, which had declined to ≤50% of the baseline value with normal renal function	–
Liang et al^[21]^	Proteinuria <0.5 g/day with stable renal function	Proteinuria 0.5–3.5 g/day, which had declined to ≤50% of the baseline value with well-preserved renal function	Proteinuria >3.5 g/day or a persistent severe hypoproteinaemia in patients who had achieved CR or PR
Peng et al^[22]^	Proteinuria <0.3 g/day with serum ALB ≥35 g/L	Serum albumin ≥30 g/L or proteinuria 0.4–3.0 g/day, which had declined to ≤50% of the baseline value	–
Liang^[23]^	Proteinuria <0.3 g/day with normal serum ALB and SCr	Proteinuria <3.5 g/day, which had declined to ≤50% of the baseline value with serum ALB elevated and stable SCr	Proteinuria >3.5 g/day in patients who had achieved CR or PR
Chen et al^[24]^	Proteinuria <0.3 g/day with normal serum ALB (>35 g/L) and renal function	Proteinuria 0.3–3.5 g/day, which had declined to ≤50% of the baseline value with stable renal function	–
Yao^[25]^	Proteinuria <0.3 g/day with normal serum ALB (>35 g/L) and renal function	A decrease of at least 50% in daily proteinuria with serum ALB elevated and stable SCr	Proteinuria >3.5 g/day in patients who had achieved CR or PR
Liu^[26]^	Proteinuria ≤0.5 g/day with serum ALB ≥35 g/L	Serum albumin ≥30 g/L and proteinuria 0.5–3.0 g/day, which had declined to ≤50% of the baseline value	–
Zhang^[27]^	Proteinuria <0.3 g/day with stable renal function	Proteinuria 0.5–3.0 g/day, which had declined to ≤50% of the baseline value with stable renal function	Proteinuria >3.0 g/day in patients who had achieved CR or PR
Hu et al^[28]^	Proteinuria <0.3 g/day with normal serum ALB (>35 g/L) and renal function	Proteinuria 0.3–3.5 g/day, which had declined to ≤50% of the baseline value with serum ALB elevated and stable renal function	Proteinuria >3.5 g/day in patients who had achieved CR or PR

ALB = albumin, CR = complete remission, PR = partial remission, SCr = serum creatinine.

### 2.5 Statistical analysis

We performed the data analysis by using Review Manager Version 5.3 (Cochrane Collaboration). Heterogeneity between studies was assessed by using *I*^2^ statistics. We considered *I*^2^ >50% and *P* < .10 to imply significant heterogeneity. Homogeneous data were analyzed using the fixed-effects model. Heterogeneous data were analyzed using the random-effects model. We presented categorical variables as odds ratios (ORs). Summary estimates and 95% confidence intervals (CIs) were calculated. Overall effects were determined by the using Z-test. A *P* value <.05 was considered significant. Publication bias was assessed using subgroup analysis and sensitivity analysis.

## 3 Results

### 3.1 Study selection and characteristics

**Figure 1:**
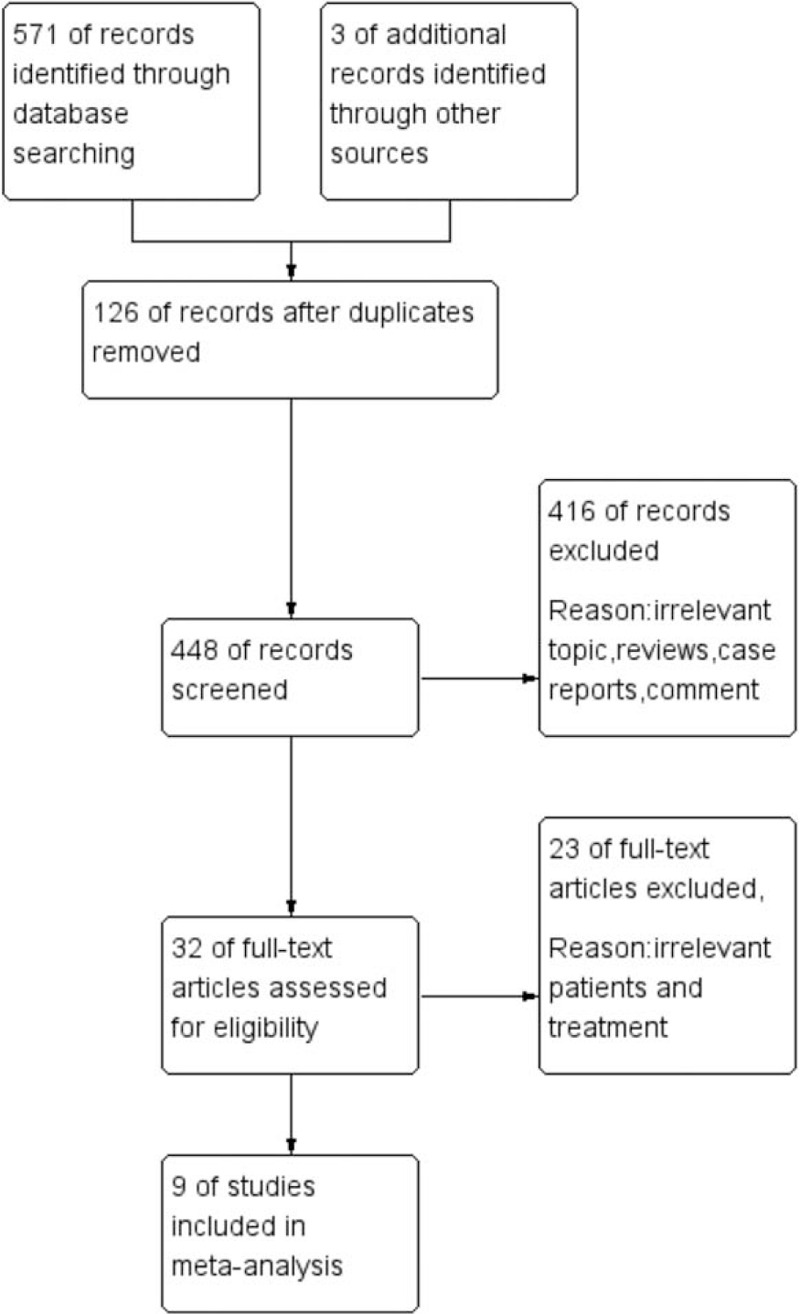
Flow diagram of the literature search.

A flow diagram of the selection process is shown in Figure 1. Ultimately, nine studies from China were included in this analysis.^[20–28]^ Of the 9 studies, 1 was an RCT, and 8 were cohort studies. Eight studies were published in Chinese journals. Overall, 228 patients were included in the TAC monotherapy group, and 214 patients were included in the CTX-steroid combination therapy group. The follow-up period was from 6 to 18 months. The risk of bias in the included RCTs was moderate. The cohort studies achieved scores of ≥6 points and were considered to be of high quality. The baseline characteristics of these studies are listed in Table 2. Specific drug treatment programs are listed in Table 3. The Cochrane assessment is listed in Table 4, and the NOS assessment is listed in Table 5.

**Table 2:** Characteristics of the included studies.

Study (year)	Country	Study design	Follow-up period	Sample size	Mean age, y	Male/female	SCr, μmol/L	Proteinuria, g/day	ACEI and/or ARB treatment
Wang, 2020^[20]^	China	Cohort study	6 mo	TAC group: 48 CTX group: 48	55 ± 1.2 56 ± 1.5	29/19 28/20	94.5 ± 33.1 99.2 ± 25.8	5.6 ± 2.6 5.0 ± 2.6	+
Liang et al, 2017^[21]^	China	Cohort study	12 mo	TAC group: 30 CTX group: 28	48.2 ± 13.5 53.9 ± 10.4	16/14 9/19	70.7 ± 17.5 81.0 ± 22.5	5.9 ± 2.7 6.9 ± 2.2	+
Peng et al, 2015^[22]^	China	Cohort study	18 mo	TAC group: 22 CTX group: 22	44.6 ± 1.6 46.6 ± 2.3	11/11 12/10	–	–	?
Liang, 2014^[23]^	China	Cohort study	6 mo	TAC group: 7 CTX group:10	58.4 ± 6.0 52.8 ± 4.9	3/4 7/3	68.4 ± 9.1 91.6 ± 16.2	–	+
Chenet al, 2019^[24]^	China	Cohort study	6 mo	TAC group: 14 CTX group:32	51.2 ± 15.7 57.6 ± 8.4	6/8 22/10	94.5 ± 33.1 99.2 ± 25.9	5.6 ± 2.6 5.2 ± 2.6	?
Yao, 2017^[25]^	China	Cohort study	6 mo	TAC group: 18 CTX group:20	48.3 ± 13.2 45.1 ± 13.6	8/10 13/7	54.2 ± 14.9 64.0 ± 15.0	4.9 ± 1.0 9.4 ± 0.8	+
Liu, 2009^[26]^	China	RCT	6 mo	TAC group: 10 CTX group:10	52.1 ± 8.4 54.1 ± 9.1	6/4 7/3	–	–	+
Zhang, 2019^[27]^	China	Cohort study	6 mo	TAC group: 45 CTX group:27	55.1 ± 11.1 55.4 ± 10.3	27/18 18/9	68.9 ± 16.8 70.3 ± 25.0	5.3 ± 2.8 6.9 ± 4.0	+
Hu et al, 2020^[28]^	China	Cohort study	12 mo	TAC group:34 CTX group:17	49.2 ± 9.9 53.4 ± 5.6	20/14 9/8	68.8 ± 19.5 72.8 ± 16.3	6.6 ± 2.8 7.7 ± 3.3	+

? = no description, + = patient was treated by ACEI and/or ARB, ACEI = angiotensin-converting enzyme inhibitor, ARB = angiotensin II subtype 1 receptor blocker, CTX = cyclophosphamide, SCr = serum creatinine, TAC = tacrolimus.

**Table 3:** Specific drug treatment program.

Study	TAC regimens	CTX regimens
Wang^[20]^	Oral TAC 0.05 mg/kg/day for the 6 mo (target trough blood concentration of 3–5 ng/mL)	IV CTX once a mo for 6 mo (accumulated dosage of 7.5–11.5 g); oral prednisone 0.5 mg/kg/day for 2 mo with gradual tapering
Liang et al^[21]^	Oral TAC 0.05–0.1 mg/kg/day (target trough blood concentration of 5–10 ng/mL for 6 mo and then 4–6 ng/mL in the subsequent 3 mo with gradual tapering)	IV CTX 0.5–0.75 g/m^2^ once a month for 6 mo and then once in every 2–3 mo; oral prednisone 1 mg/kg/day for 1 mo with gradual tapering
Penget al^[22]^	Oral TAC 0.05–0.1 mg/kg/day (the trough blood concentration of 5–10 ng/mL for 18 mo)	IV CTX 0.8–1.0 g once a mo for 6 mo and then once in every 2–3 mo; oral prednisone 1 mg/kg/day for 2–3 mo with gradual tapering
Liang^[23]^	Oral TAC 0.05 mg/kg/day (the trough blood concentration of 4–10 ng/mL for 6 mo)	IV CTX 0.8–1.2 g once in every 2 wk for 2 mo and then once a mo; oral prednisone initial dose of 0.5 mg/kg/day
Chen et al,^[24]^	Oral TAC 0.05 mg/kg/day (the trough blood concentration of 3–8 ng/mL for 6 mo)	IV CTX 0.5–0.75 g/m^2^ once a mo for 6 months; oral prednisone 0.5 mg/kg/day for 2 mo with gradual tapering
Yao^[25]^	Oral TAC 0.05 mg/kg/day (the trough blood concentration of 4–10 ng/mL for 6 mo)	IV CTX 1 g once a mo for 6 mo; oral prednisone 0.8–1 mg/kg/day with gradual tapering
Liu^[26]^	Oral TAC 0.05 mg/kg/day (the trough blood concentration of 5–10 ng/mL for 6 mo)	IV CTX 0.6g once in every 2 wk for 3 mo and then 1g once a mo; oral prednisone oral prednisone 1 mg/kg/day for 2 mo with gradual tapering
Zhang^[27]^	Oral TAC 0.1 mg/kg/day (the trough blood concentration of 5–10 ng/mL for 6 mo)	IV CTX 0.75 g/m^2^ monthly and then once in every 2–3 mo (accumulated dosage was <10 g); oral prednisone 1 mg/kg/day for 2 mo with gradual tapering
Huet al^[28]^	Oral TAC 0.05 mg/kg/day (the trough blood concentration of 5–10 ng/mL for 6 mo then 4–6 ng/mL in case of remission)	Oral CTX 100 mg/day (accumulated dosage of 8–12 g); oral prednisone 0.5 mg/kg/day for 2 mo with gradual tapering

CTX = cyclophosphamide, TAC = tacrolimus.

**Table 4:** Quality assessment of randomized control trial.

Study	Random sequence generation	Allocation concealment	Blinding of participants and personnel	Incomplete outcome data	Selective reporting	Other bias
Liu^[26]^	?	?	?	+	+	?

The randomized control trial was evaluated using the Cochrane assessment tool. + = low risk of bias, ? = unclear risk of bias.

**Table 5:** Quality assessment of cohort studies.

Studies	Selection	Comparability	Outcome	Score
Wang^[20]^	★★★	★	★★	6
Liang et al^[21]^	★★★★	★	★★★	8
Peng et al^[22]^	★★★	★	★★	6
Liang^[23]^	★★★★	★	★★★	8
Chen et al^[24]^	★★★	★	★★★	7
Yao^[25]^	★★★★	★	★★★	8
Zhang^[27]^	★★★★	★	★★★	8
Huet al^[28]^	★★★★	★	★★★	8

The Cohort studies were evaluated using the Newcastle-Ottawa scale, which are comprised of the study of selection (Representativeness of the exposed group, Representativeness of the non exposed group, Ascertainment of exposure, Demonstration that outcome of interest was not present at start of study), group comparability(Controls for the most important factor, Controls for any additional factor), outcome measures (Assessment of outcome, Was follow-up long enough for outcomes to occur, Adequacy of follow up of cohorts), a total of 9 points. ★, 1 point.

### 3.2 Meta-analysis results

#### 3.2.1 CR at month six

**Figure 2:**
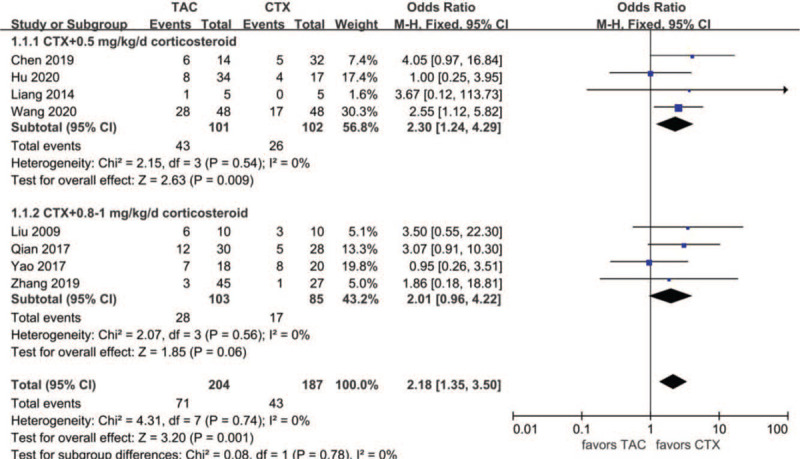
Forest plots comparing CR at the sixth month between TAC and CTX. CR = complete remission, CTX = cyclophosphamide, TAC = tacrolimus.

Data comparing TAC with CTX combined with corticosteroids at 0.5 mg/kg/day concerning CR at month 6 were reported in four articles: 43 of 101 (42.6%) for the TAC group and 26 of 102 (25.5%) for the CTX group. The heterogeneity between the 2 studies was not substantial (*P* = .54, *I*^2^ = 0%), so the fixed-effects model was used for the meta-analysis. CR at month 6 was higher in the TAC group than in the CTX combined with corticosteroids at 0.5 mg/kg/day group (OR 2.30, 95% CI 1.24–4.29, *P* < .01). Data comparing CR for TAC with CTX combined with corticosteroids at 0.8–1 mg/kg/day at month 6 were reported in 4 articles: 28 of 103 (27.2%) for the TAC group and 17 of 85 (20%) for the CTX group. The heterogeneity between the2 studies was not substantial (*P* = .56, *I*^2^ = 0%), so the fixed-effects model was used for the meta-analysis. CR at month 6 was higher in the TAC group than in the CTX combined with corticosteroids at 0.8 to 1 mg/kg/day group, but the difference was not statistically significant (OR 2.01, 95% CI 0.96–4.22, *P* = .06). There was no significant difference between the 2 subgroups (*P* = .78). As a whole, CR at month 6 was higher in the TAC group than in the CTX group (OR 2.18, 95% CI 1.35–3.50, *P* < .01) (Fig. 2).

#### 3.2.2 PR at month 6

**Figure 3:**
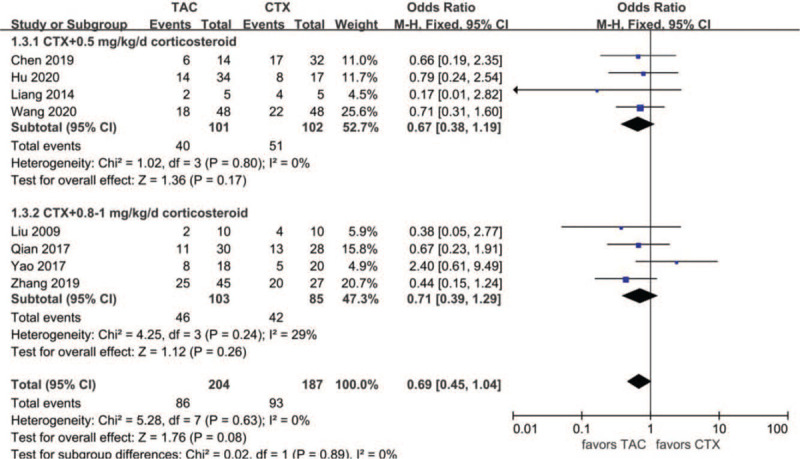
Forest plots comparing PR at the 6th month between TAC and CTX. CTX = cyclophosphamide, PR = partial remission, TAC = tacrolimus.

Data about PR at month six were reported in eight articles: 86 of 204 (42.2%) for the TAC group and 93 of 187 (49.7%) for the CTX group. The heterogeneity between the 2 studies was not substantial (*P* = .63, *I*^2^ = 0%), so the fixed-effects model was used for the meta-analysis. PR at month 6 was lower in the TAC group than in the CTX group, but the difference was not statistically significant (OR 0.69, 95% CI 0.45–1.04, *P* = .08). There was no significant difference between the 2 subgroups (*P* = .89) **(**Fig. 3).

#### 3.2.3 TR at month 6

**Figure 4:**
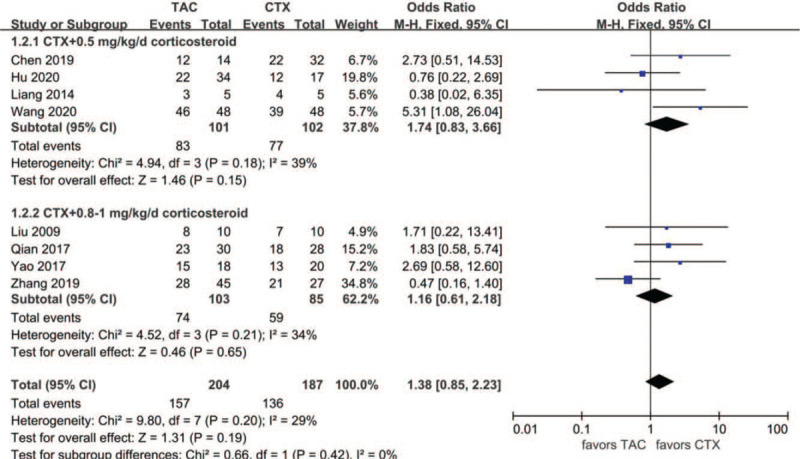
Forest plots comparing TR at the 6th month between TAC and CTX. CTX = cyclophosphamide, TAC = tacrolimus, TR = total remission.

Data about TR at month six were reported in eight articles: 157 of 204 (77.0%) for the TAC group and 136 of 187 (72.7%) for the CTX group. The heterogeneity between the 2 studies was not substantial (*P* = .20, *I*^2^ = 29%), so the fixed-effects model was used for the meta-analysis. TR at month 6 was higher in the TAC group than in the CTX group, but the difference was not statistically significant (OR 1.38, 95% CI 0.85–2.23, *P* = .19). There was no significant difference between the 2 subgroups (*P* = .42) **(**Fig. 4).

#### 3.2.4 CR after 1 year

**Figure 5:**
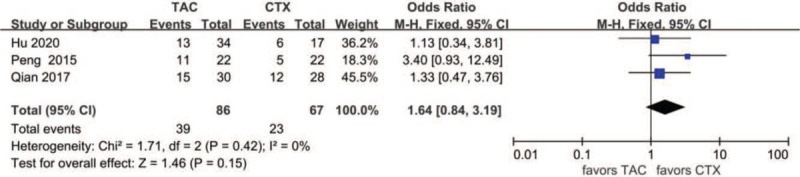
Forest plots comparing CR after 1 year between TAC and CTX. CR = complete remission, CTX = cyclophosphamide, TAC = tacrolimus.

Data about CR after 1 year were reported in 3 articles: 39 of 86(45.3%) for the TAC group and 23 of 67 (34.3%) for the CTX group. The heterogeneity between the 2 studies was not substantial (*P* = .42, *I*^2^ = 0%), so the fixed-effects model was used for the meta-analysis. CR after 1 year was higher in the TAC group than in the CTX group, but the difference was not statistically significant (OR 1.64, 95% CI 0.84–3.19, *P* = .15) (Fig. 5).

#### 3.2.5 PR after 1 year

**Figure 6:**
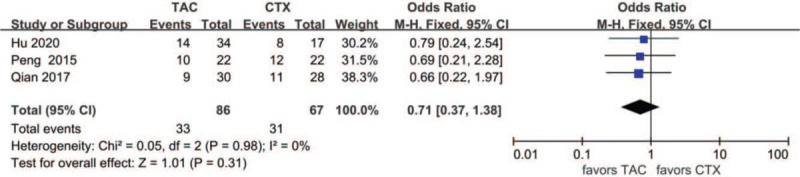
Forest plots comparing PR after 1 year between TAC and CTX. CTX = cyclophosphamide, PR = partial remission, TAC = tacrolimus.

Data about PR after 1 year were reported in 3 articles: 33 of 86 (38.4%) for the TAC group and 31 of 67 (46.3%) for the CTX group. The heterogeneity between the 2 studies was not substantial (*P* = 0.98, *I*^2^ = 0%), so the fixed-effects model was used for the meta-analysis. PR after 1 year was lower in the TAC group than in the CTX group, but the difference was not statistically significant (OR 0.71, 95% CI 0.37–1.38, *P* = .31) (Fig. 6).

#### 3.2.6 TR after 1 year

**Figure 7:**
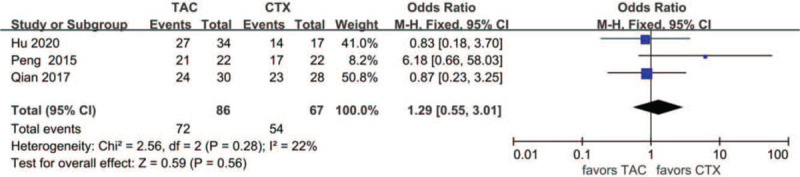
Forest plots comparing TR after 1 year between TAC and CTX. CTX = cyclophosphamide, TAC = tacrolimus, TR = total remission.

Data about TR after 1 year were reported in 3 articles: 72 of 86 (83.7%) for the TAC group and 54 of 67 (80.6%) for the CTX group. The heterogeneity between the 2 studies was not substantial (*P* = .28, *I*^2^ = 22%), so the fixed-effects model was used for the meta-analysis. There was no significant difference between the 2 groups concerning TR after 1 year (OR 1.29, 95% CI 0.55–3.01, *P* = .56) (Fig. 7).

#### 3.2.7 Relapse rate

**Figure 8:**
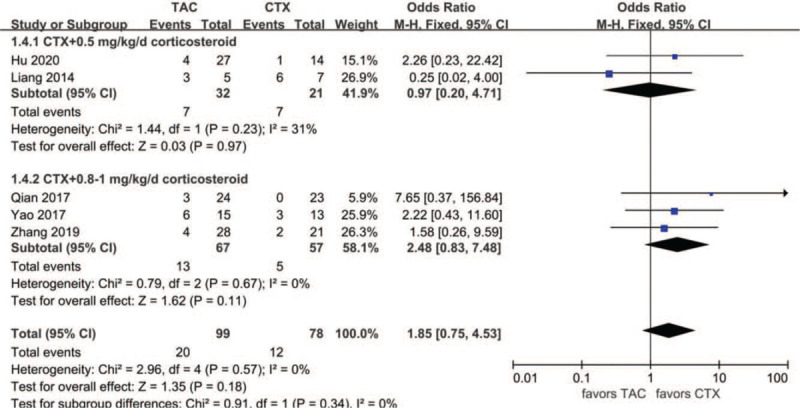
Forest plots comparing relapse rate between TAC and CTX. CTX = cyclophosphamide, TAC = tacrolimus.

Data about relapse rate were reported in 5 articles: 22 of 99 (22.2%) for the TAC group and 12 of 78 (15.4%) for the CTX group. The heterogeneity between the 2 studies was not substantial (*P* = .57, *I*^2^ = 0%), so the fixed-effects model was used for the meta-analysis. The relapse rate was higher in the TAC group than in the CTX group, but the difference was not statistically significant (OR 1.85, 95% CI 0.75–4.53, *P* = .18). Subgroup analysis showed that the relapse rate was lower in the TAC group than in the CTX combined with corticosteroids at 0.5 mg/kg/day group, but the difference was not statistically significant (*P* > .05). In addition, subgroup analysis showed that the relapse rate was higher in the TAC group than in the CTX combined with corticosteroids at 0.8 to 1 mg/kg/day group, but the difference was not statistically significant (*P* > .05) (Fig. 8).

#### 3.2.8 Drug-related adverse effects

**Figure 9:**
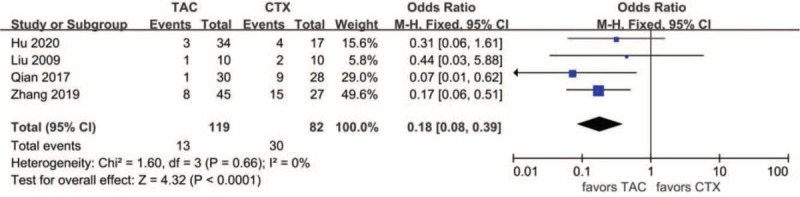
Forest plots comparing infection between TAC and CTX. CTX = cyclophosphamide, TAC = tacrolimus.

**Figure 10:**
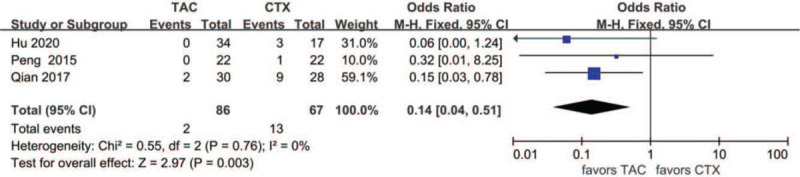
Forest plots comparing leukopenia between TAC and CTX. CTX = cyclophosphamide, TAC = tacrolimus.

**Figure 11:**
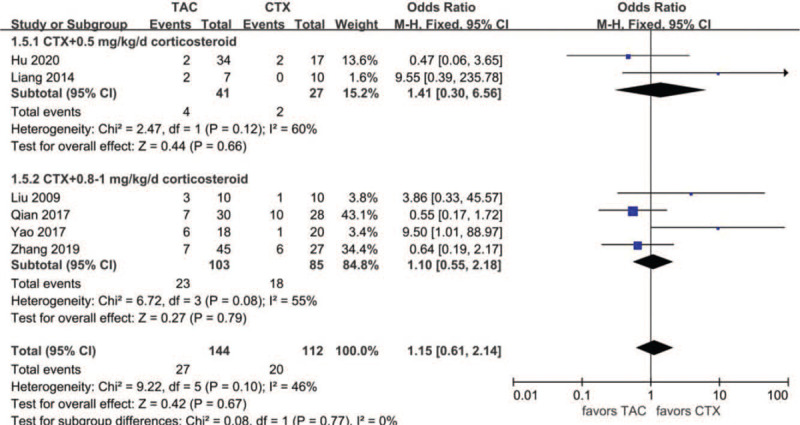
Forest plots comparing glucose intolerance between TAC and CTX. CTX = cyclophosphamide, TAC = tacrolimus.

**Figure 12:**
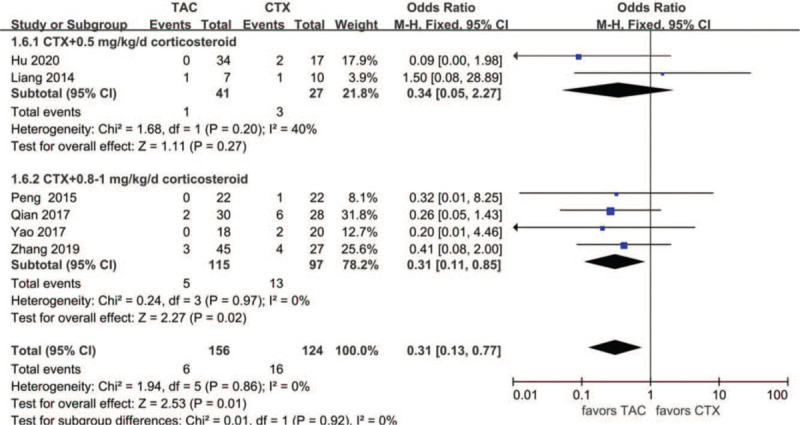
Forest plots comparing abnormal aminotransferase between TAC and CTX. CTX = cyclophosphamide, TAC = tacrolimus.

**Figure 13:**
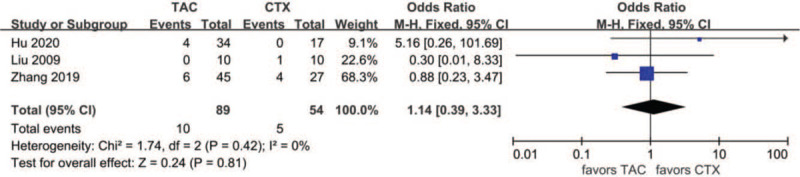
Forest plots comparing acute renal failure between TAC and CTX. CTX = cyclophosphamide, TAC = tacrolimus.

**Figure 14:**
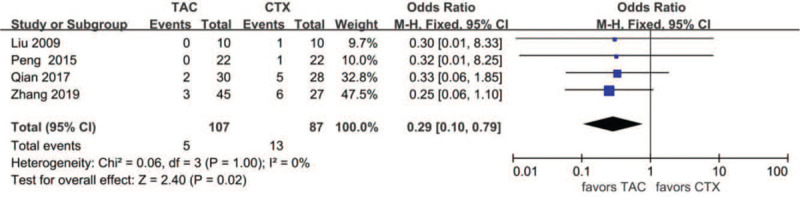
Forest plots comparing gastrointestinal symptoms between TAC and CTX. CTX = cyclophosphamide, TAC = tacrolimus.

**Figure 15:**
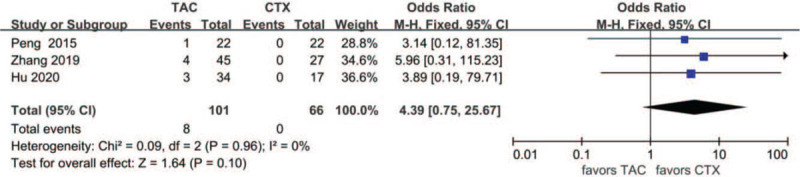
Forest plots comparing tremor between TAC and CTX. CTX = cyclophosphamide, TAC = tacrolimus.

Data about drug-related adverse effects were reported in seven articles. The incidences of infection (10.9%, 13/119), leukopenia (2.3%, 2/86), glucose intolerance (18.8%, 27/144), abnormal aminotransferase (3.8%, 6/156), acute renal failure (11.2%, 10/89), gastrointestinal symptoms (4.7%, 5/107), and tremors (7.9%, 8/101) were reported in the TAC group. The incidences of infection (36.6%, 30/82), leukopenia (19.4%, 13/67), glucose intolerance (17.9%, 20/112), abnormal aminotransferase (12.9%, 16/124), acute renal failure (9.3%, 10/89), gastrointestinal symptoms (14.9%, 13/87), and tremors (0%, 0/66) were reported in the CTX group. There was no statistically significant difference between the 2 groups concerning glucose intolerance (OR 1.15, 95% CI 0.61–2.14, *P* = .67), acute renal failure (OR 1.14, 95% CI 0.39–3.33, *P* = .81), or tremors (OR 4.39, 95% CI 0.75–25.67, *P* = .10). Incidences of gastrointestinal symptoms (OR 0.29, 95% CI 0.10–0.79, *P* = .02), infection (OR 0.18, 95%CI 0.08–0.39, *P* < .01), leukopenia (OR 0.14, 95% CI 0.04–0.51, *P* < .01), and abnormal aminotransferase (OR 0.31, 95% CI 0.13–0.77, *P* = .01) were all lower in the TAC group than in the CTX group. There was no significant difference between the 2 subgroups concerning glucose intolerance (*P* > .05). In addition, subgroup analysis showed that there was no significant difference between the TAC group and the CTX combined with corticosteroids at 0.5 mg/kg/day group concerning abnormal aminotransferase (*P* > .05). All forest plots of drug-related adverse effects are provided in Figures 9–15.

### 3.3 Sensitivity analyses

Some outcomes did not require subgroup analysis, so sensitivity analyses were used to judge the reliability of the results. We removed 1 study at a time, and the results of the meta-analysis still showed no difference.

## 4 Discussion

IMN is considered damage to glomerular podocytes mediated by autoantibodies.^[29]^ TAC, a calcineurin inhibitor (CNI), mainly binds to a particular intracellular receptor called FK-506-binding protein 12 to inhibit calcineurin phosphatase, thereby inhibiting cytokines such as interleukin-2. Consequently, TAC can inhibit the growth and differentiation of T cells, thereby reducing the immune damage of podocytes.^[30]^ Our meta-analysis revealed that TAC monotherapy had a higher CR at month six compared with CTX-corticosteroid combination therapy for IMN. However, after 1 year, the 2 therapy regimens had a similar CR, PR, TR, and relapse rate. Moreover, TAC monotherapy had fewer side effects concerning infection, gastrointestinal symptoms, leukopenia, and abnormal aminotransferase.

Sustained remission of nephrotic syndrome is very important for patients with IMN and can reduce complications and prevent progression to end-stage renal disease. Our study found that TAC monotherapy or CTX-corticosteroid combination therapy had similar remission rates, and both had a high remission rate of nearly 80%. However, our meta-analysis found that IMN patients achieved CR more rapidly using TAC monotherapy at month six. A previous meta-analysis also showed that TAC combined with corticosteroids had a significantly higher remission rate than CTX combined with corticosteroids at the early stage, but no significant difference was observed at the longest follow-up periods.^[17,31]^ CTX was slow to take effect because cyclophosphamide usually requires a cumulative dose to impart an immunosuppressive effect.^[24]^ In addition, compared with CTX combined with moderate-dose corticosteroids at 0.5 mg/kg/day, CTX combined with large-dose corticosteroids at 0.8 to 1 mg/kg/day did not produce a higher remission rate.

Low relapse rate is beneficial for IMN patients. Many studies have shown that IMN patients tend to relapse after CNI tapering or withdrawal, and the relapse rate ranges from 13% to 50%.^[32]^ A study by Praga et al^[33]^ revealed that the relapse rate after TAC withdrawal was 47%, with no significant difference in the placebo group. A previous meta-analysis showed that the relapse rate of TAC combined with corticosteroids was not different from that of CTX-corticosteroid combination therapy.^[17,31]^ Our study observed that the relapse rate after remission with TAC monotherapy was 22.2%. TAC monotherapy tended to have a higher relapse rate, but the difference was not significant compared with CTX-corticosteroid combination therapy. There are low levels of evidence that prolonged TAC treatment with a low blood concentration is beneficial to sustained remission and reducing the relapse rate. Subgroup analysis suggested that CTX combined with large-dose corticosteroids at 0.8 to 1 mg/kg/day had the lowest relapse rate (8.8%), but there was no significant difference compared with TAC.

Long-term use of immunosuppressive therapy can increase the incidence of drug-related adverse effects, so clinicians should evaluate the beneficial and adverse effects when prescribing treatment regimens for IMN patients. The nephrotoxicity of CNI is an important issue, which has limited its applications. Many studies have attempted to investigate the nephrotoxicity of CNIs. In recent years, an RCT showed that TAC had no significant nephrotoxicity.^[34]^ Our study observed that the incidence of acute renal failure in the TAC group was 11.2%, which was slightly higher than that in the CTX group, with no significant difference. TAC and corticosteroids can both raise blood glucose. Our study observed that the incidence of glucose intolerance was not different between TAC monotherapy and CTX-corticosteroid combination therapy. TAC can cause tremors. Our study observed that none of the IMN patients using CTX had tremors, but the difference was not significant compared with TAC, which needs to be further verified. Moreover, our study observed that TAC monotherapy had fewer side effects concerning infection, gastrointestinal symptoms, leukopenia, and abnormal aminotransferase, which showed the advantages of TAC monotherapy. Furthermore, subgroup analysis suggested that CTX combined with large-dose corticosteroids at 0.8 to 1 mg/kg/day did not produce a higher remission rate but may have produced a higher incidence of drug-related adverse effects, such as abnormal aminotransferase.

There were some limitations in our meta-analysis. First, IMN has the possibility of spontaneous remission.^[35]^ Kidney Disease Improving Global Outcomes guidelines suggest that immunosuppressive therapy can be given to IMN patients with urine protein >4 g/24 hours and no decrease in urine protein after 6 months of conservative treatment.^[11]^ However, in the majority of studies, immunosuppressants were used as soon as IMN was detected without 6 months of conservative treatment. Second, in some included studies, IMN patients were treated with angiotensin-converting enzyme inhibitor or angiotensin II subtype 1 receptor blocker (ARB) antihypertensive drugs. Since angiotensin-converting enzyme inhibitor and angiotensin II subtype 1 receptor blocker drugs have the effect of reducing proteinuria, they will affect the interference results. Third, there is no detailed definition of adverse drug reactions. Fourth, in the included studies, there were some differences concerning the specific drug regimen and definition of outcomes, which may cause a risk of bias. The target trough blood concentration of the TAC group was generally 3 to 10 ng/mL. The cumulative dose of CTX was generally 6 to 8 g and did not exceed 12 g. In some studies, the CTX group was combined with moderate-dose corticosteroids at 0.5 mg/kg/day; in other studies, the CTX group was combined with large-dose corticosteroids at 0.8 to 1 mg/kg/day. Therefore, we performed subgroup analysis according to the dosage of different corticosteroids, which can reduce risk bias.

## 5 Conclusions

Our meta-analysis revealed that TAC monotherapy is comparable to CTX-corticosteroid combination therapy for renal remission in IMN patients. TAC monotherapy had a higher CR in the early stage and had fewer drug-related adverse effects. The relapse rate of TAC monotherapy was higher than that of CTX-corticosteroid combination therapy, but the difference was not significant. To further confirm this conclusion, more large multicenter randomized controlled trials comparing the 2 drug treatment regimens are necessary.

## Acknowledgments

All authors give permission to be named.

## Author contributions

**Conceptualization:** Wei Xu, Lifeng Gong, and Min Xu.

**Data curation:** Wei Xu, Weigang Tang, Wei Jiang, Fengyan Xie, Liping Ding.

**Investigation:** Lifeng Gong, Min Xu, and Jingkui Lu.

**Methodology:** Lifeng Gong, Min Xu, Wei Xu.

**Software:** Wei Xu, Weigang Tang, and Jingkui Lu.

**Supervision:** Lifeng Gong, Min Xu, Wei Xu.

**Writing – original draft:** Wei Xu, Wei Jiang, Fengyan Xie, Liping Ding, Xiaoli Qian.

**Writing – review & editing:** Wei Xu, Wei Jiang, Liping Ding, Fengyan Xie, and Xiaoli Qian.

## References

[1] du Buf-Vereijken PWG Branten AJW Wetzels JFM. Idiopathic membranous nephropathy:outline and rationale of a treatment strategy. Am J Kidney Dis 2005;46:1012–29. 16310567 10.1053/j.ajkd.2005.08.020

[2] Haas M Meehan SM Karrison TG. Changing etiologies of unexplained adult nephrotic syndrome: a comparison of renal biopsy findings from 1976-1979 and 1995-1997. Am J Kidney Dis 1997;30:621–31. 9370176 10.1016/s0272-6386(97)90485-6

[3] Glassock RJ. The pathogenesis of idiopathic membran ous nephropathy: a 50-year odyssey. Am J Kidney Dis 2010;56:157–67. 20378220 10.1053/j.ajkd.2010.01.008

[4] Hladunewich MA Troyanov S Calafati J. The natural history of the non nephrotic membranous nephropathy patient. Clin J Am Soc Nephrol 2009;4:1417–22. 19661220 10.2215/CJN.01330209 PMC2736692

[5] Glassock RJ. Diagnosis and natural course of membranous nephropathy. Semin Nephrol 2003;23:324–32. 12923720 10.1016/s0270-9295(03)00049-4

[6] Schieppati A Mosconi L Perna A. Prognosis of untreated patients with idiopathic membranous nephropathy. N Engl J Med 1993;329:85–9. 8510707 10.1056/NEJM199307083290203

[7] Kidney Disease: Improving Global Outcomes (KDIGO) Glomerulonephritis Work Group. Chapter 7: Idiopathic membranous nephropathy. Kidney Int Suppl 2012;2:186–97. 10.1038/kisup.2012.20 PMC4089709 25018932

[8] Knight A Askling J Granath F. Urinary bladder cancer in Wegener's granulomatosis: risks and relation to cyclophosphamide. Ann Rheum Dis 2004;63:1307–11. 15130900 10.1136/ard.2003.019125 PMC1754772

[9] van den Brand JAJG van Dijk PR Hofstra JM Wetzels JFM. Cancer risk after cyclophosphamide treatment in idiopathic membranous nephropathy. Clin J Am Soc Nephrol 2014;9:1066–73. 24855280 10.2215/CJN.08880813 PMC4046727

[10] Faurschou M Sorensen IJ Mellemkjaer L. Malignancies in Wegener's granulomatosis: incidence and relation to cyclophos phamide therapy in a cohort of 293 patients. J Rheumatol 2008;35:100–5. 17937462

[11] Eknoyan G. KDIGO Clinical Practice Guideline for Glomerulonephritis. Kidney Int Suppl 2012;2:143–53.

[12] Artz MA Boots JM Ligtenberg G. Conversion from cyclosporine to tacrolimus improves quality of life indices, renal graft function and cardiovascular risk profile. Am J Transplant 2004;4:937–45. 15147428 10.1111/j.1600-6143.2004.00427.x

[13] Jardine AG. Assessing the relative risk of cardiovascu lar disease among renal transplant patients receiving tacrolimus or cyclosporine. Transpl Int 2005;18:379–84. 15773954 10.1111/j.1432-2277.2005.00080.x

[14] Grimm M Rinaldi M Yonan NA. Superior prevention of acute rejection by tacrolimus vs. cyclosporine in heart transplant recipients-a large European trial. Am J Transplant 2006;6:1387–97. 16686762 10.1111/j.1600-6143.2006.01300.x

[15] Choudhry S Bagga A Hari P. Effificacy and safety of tacrolimus versus cyclosporine in children with steroid-resistant nephrotic syndrome: a randomized controlled trial. Am J Kidney Dis 2009;53:760–9. 19268410 10.1053/j.ajkd.2008.11.033

[16] Li YC Huang J Li X Zhao SM. A comparison of cyclophosphamide versus tacrolimus in terms of treatment effect for idiopathic membranous nephropathy: a meta-analysis. Nefrologia 2019;39:269–76. 30755327 10.1016/j.nefro.2018.10.008

[17] Zhu LB Liu LL Yao L. Efficacy and safety of tacrolimus versus cyclophosphamide for primary membranous nephropathy: a meta-analysis. Drugs 2017;77:187–99. 28084563 10.1007/s40265-016-0683-z

[18] Furlan AD Malmivaara A Chou R. 2015 Updated method guideline for systematic reviews in the Cochrane back and neck group. Spine (Phila Pa 1976) 2015;40:1660–73. 26208232 10.1097/BRS.0000000000001061

[19] Stang A. Critical evaluation of the Newcastle-Ottawa scale for the assessment of the quality of nonrandomized studies in meta-analyses. Eur J Epidemiol 2010;25:603–5. 20652370 10.1007/s10654-010-9491-z

[20] Wang YH. Clinical observation of tacrolimus monotherapy in idiopathic membranous nephropathy. Clinical Journal of Diabetes World 2020;17:62.

[21] Liang Q Li H Xie X Qu F. The efficacy and safety of tacrolimus monotherapy in adult-onset nephrotic syndrome caused by idiopathic membranous nephropathy. Ren Fail 2017;39:512–8. 28562168 10.1080/0886022X.2017.1325371 PMC6014322

[22] Peng YH Xiao J Yu XM. Effect valuation of treatment to idiopathic membranous nephropathy of 22 cases by Tacrolimus. J Front Med 2015;5:31–2.

[23] Liang LD. Tacrolimus Monotherapy in Treatment of Nephrotic Idiopathic Membranous Nephropathy. Master's thesis of Zhejiang University 2014;01–43.

[24] Chen Q Min JJ Dai ZQ. Clinical efficacy of tacrolimus monotherapy in the treatment of idiopathic membranous nephropathy. Guangdong Medical Journal 2019;40:2774–81.

[25] Yao ZE. Four kinds of immunosuppressant therapy on the clinical effect of the treatment of idiopathic membranous nephropathy. Master's thesis of China Medical University 2017;01–34.

[26] Liu JP. The Study on the Treatment of Idiopathic Membranous Nephropathy with Tacrolimus. Master's thesis of China Medical University 2009;01–25.

[27] Zhang XX. Comparison of the efficacy of tacrolimus monotherapy,tacrolimus combined with hormone,cyclophosphamide combined with hormone in the treatment of idiopathic membaanous. Master's thesis of Tianjin Medical University 2019;01–40.

[28] Hu XY Wang LW Yang ZJ. Clinical analysis of tacrolimus monotherapy in treating idiopathic membranous nephropathy. Henan Med Res 2020;29:995–9.

[29] Debiec H Guigonis V Mougenot B. Antenatal membranous glomerulonephritis due to anti-neutral endopeptidase antibodies. N Engl J Med 2002;346:2053–60. 12087141 10.1056/NEJMoa012895

[30] Rauch MC San Martín A Ojeda D. Tacrolimus causes a blockage of protein secretion which reinforces its immunosuppressive activity and also explains some of its toxic side-effects. Transpl Immunol 2009;22:72–81. 19628039 10.1016/j.trim.2009.07.001

[31] Lin W Li HY Lin S. Efficacy and safety of tacrolimus vs cyclophosphamide in the therapy of patients with idiopathic membranous nephropathy: a meta-analysis. Drug Des Devel Ther 2019;13:2179–86. 10.2147/DDDT.S209211 PMC6613398 31308629

[32] Jha V Ganguli A Saha TK. A randomized, controlled trial of steroids and cyclophosphamide in adults with nephrotic syndrome caused by idiopathic membranous nephropathy. J Am Soc Nephrol 2007;1:1899–904. 10.1681/ASN.2007020166 17494881

[33] Praga M Barrio V Juarez GF. Tacrolimus monotherapy in membranous nephropathy: a randomized controlled trial. Kidney Int 2007;71:924–30. 17377504 10.1038/sj.ki.5002215

[34] Zen Y Onodera M Inoue D. Retroperitoneal fibrosis: a clinicopathologic study with respect to immunoglobulin G4. Am J Surg Pathol 2009;33:1833–9. 19950407 10.1097/pas.0b013e3181b72882

[35] Schieppati A Mosconi L Perna A. Prognosis of patients with idiopathic membranous nephropathy. N Engl J Med 1993;329:85–9. 8510707 10.1056/NEJM199307083290203

